# Early systemic insults following severe sepsis-associated encephalopathy of critically ill patients: association with mortality and awakening—an analysis of the OUTCOMEREA database

**DOI:** 10.1186/s40560-024-00773-9

**Published:** 2025-01-14

**Authors:** Michael Thy, Romain Sonneville, Stéphane Ruckly, Bruno Mourvillier, Carole Schwebel, Yves Cohen, Maité Garrouste-Orgeas, Shidasp Siami, Cédric Bruel, Jean Reignier, Elie Azoulay, Laurent Argaud, Dany Goldgran-Toledano, Virginie Laurent, Claire Dupuis, Julien Poujade, Lila Bouadma, Etienne de Montmollin, Jean-François Timsit, on the behalf of the OUTCOME R. E. A. network

**Affiliations:** 1 Medical and Infectious Diseases, ICU, Hospital Bichat-Claude Bernard, Université Paris Cité, AP-HP, Paris, France; 2 UMR 1343, Pharmacology and Therapeutic Evaluation in Children and Pregnant Women, INSERM, Université Paris Cité, Paris, France; 3 UMR 1137, INSERM, Université Paris Cité, Paris, France; 4 Intensive Care Unit, CHU Reims-Champagne Clinic, Reims, France; 5 Medical Intensive Care Unit, C.H.U de Grenoble, La Tronche, France; 6 Respiratory Department, Avicenne Hospital (AP-HP), Bobigny, France; 7 Intensive Care Unit, Hospital Franco-Britannique, Fondation Cognacq-Jay, Levallois-Perret, France; 8 Réanimation, Hospital Center Sud Essonne, Étampes, France; 9 Medical and Surgical Intensive Care Unit, Hospital Paris Saint-Joseph, Paris, France; 10 Intensive Care Unit, Nantes University Hospital Hotel-Dieu, Nantes, France; 11 Medical Intensive Care Unit, Hospital Saint-Louis, Paris, France; 12 Intensive Care Unit, Hospital Édouard Herriot, Lyon, France; 13 Intensive Care Unit, Intercommunal Hospital Group Le Raincy Montfermeil, Montfermeil, France; 14 Intensive Care Unit, C.H. de Versailles, Le Chesnay, France; 15 Intensive Care Unit, CHU Gabriel-Montpied, Clermont-Ferrand, France; 16 OUTCOME REA Network, Drancy, France

**Keywords:** Critical care, Secondary brain injury, Early systemic insults, Sepsis, Encephalopathy

## Abstract

**Background:**

Sepsis-associated encephalopathy (SAE) may be worsened by early systemic insults. We aimed to investigate the association of early systemic insults with outcomes of critically ill patients with severe SAE.

**Methods:**

We performed a retrospective analysis using data from the French OUTCOMEREA prospective multicenter database. We included patients hospitalized in intensive care unit (ICU) for at least 48 h with severe SAE (defined by a score on the Glasgow Coma Scale (GCS) ≤ 13 and severe sepsis or septic shock (SEPSIS 2.0 criteria)) requiring invasive ventilation and who had no primary brain injury.

We analyzed early systemic insults (abnormal glycemia (< 3 mmol/L or ≥ 11 mmol/L), hypotension (diastolic blood pressure ≤ 50 mmHg), temperature abnormalities (< 36 °C or ≥ 38.3 °C), anemia (hematocrit < 21%), dysnatremia (< 135 mmol/L or ≥ 145 mmol/L), oxygenation abnormalities (PaO_2_ < 60 or > 200 mmHg), carbon dioxide abnormalities (< 35 mmHg or ≥ 45 mmHg), and the impact of their correction at day 3 on day-28 mortality and awakening, defined as a recovery of GCS > 13.

**Results:**

We included 995 patients with severe SAE, of whom 883 (89%) exhibited at least one early systemic insult that persisted through day 3. Compared to non-survivors, survivors had significantly less early systemic insults (hypoglycemia, hypotension, hypothermia, and anemia) within the first 48 h of ICU admission. The absence of correction of the following systemic insults at day 3 was independently associated with mortality: blood pressure (adjusted hazard ratio (aHR) = 1.77, 95% confidence interval (CI) 1.34–2.34), oxygenation (aHR = 1.78, 95% CI 1.20–2.63), temperature (aHR = 1.46, 95% CI 1.12–1.91) and glycemia (aHR = 1.41, 95% CI 1.10–1.80). Persistent abnormal blood pressure, temperature and glycemia at day 3 were associated with decreased chances of awakening.

**Conclusions:**

In patients with severe SAE, the persistence of systemic insults within the first three days of ICU admission is associated with increased mortality and decreased chances of awakening.

**Supplementary Information:**

The online version contains supplementary material available at 10.1186/s40560-024-00773-9.

## Key points

Question: Sepsis-associated encephalopathy (SAE) may be worsened by early systemic insults. We aimed to investigate the association of early systemic insults with outcomes of critically ill patients with severe SAE.

Findings: In patients with a severe sepsis-associated encephalopathy, early systemic insults were frequent and failure to correct them (especially blood pressure, oxygenation, temperature and glycemia) within the first 3 days of ICU admission was associated with increased mortality and decreased chances of awakening at day 28.

Meaning: Early correction of systemic insults in patients with SAE may be one of the targets for better outcomes.

**Supplementary Information:**

The online version contains supplementary material available at 10.1186/s40560-024-00773-9.

## Introduction

Approximately 50% of sepsis cases develop an acute encephalopathy (sepsis-associated encephalopathy, SAE) ranging from delirium to coma, mainly diagnosed with altered Glasgow Coma Scale (GCS), which is associated with increased mortality and long-term sequelae in survivors [1]. The pathophysiology of SAE involves a complex interplay of inflammatory and immune responses, oxidative stress, blood–brain barrier disruption, and hypoperfusion, resulting in brain dysfunction and neurological symptoms [2].

Several early systemic insults have been identified as risk factors for poor outcome in patients with cerebrovascular disease and after cardiac arrest, such as hypo- and hypercapnia [3, 4] and hypo- and hyperoxia [5]. Guidelines to control early systemic insults have been proposed in the post-resuscitation care guidelines [6], in neurocritical care guidelines for brain injury [7] and in patients on ECMO [8, 9]. In sepsis, early systemic insults are common and associated with worse outcomes [10–13], especially for hypo- and hyperglycemia, hypercapnia, and hypernatremia, as identified in previous studies [1, 14].

Current literature shows that a higher mean arterial pressure (MAP) target (≥ 80 mmHg) was not associated with a reduced risk of SAE or significant differences in mortality at 28 or 90 days in patients with septic shock [15, 16], but was associated with higher Richmond Agitation-Sedation Scale (RASS) during Intensive Care Unit (ICU) stay [17]. Conservative oxygen therapy (SpO_2_ between 90 and 97%) did not affect ventilator-free days [18], whereas hyperoxia in sepsis (PaO_2_ > 300 mmHg) has been associated with increased mortality [19]. Hypercapnia (> 45 mmHg) has been associated with an increased risk of SAE [1]. A recent retrospective study showed an association between PaCO_2_ during the first 24 h and all-cause mortality risk (30-day, 60-day, and 90-day) for patients with SAE in the ICU, suggesting a range of 35–50 mmHg) of PaCO_2_ as the optimal target [20]. A temperature above 38.4 °C was associated with higher mortality in a post hoc analysis of a study on lowering temperature in septic shock [21, 22]. Hypernatremia (> 145 mmol/L), hypoglycemia (< 3 mmol/l) and hyperglycemia (> 10 mmol/l) were associated with an increased risk of SAE [1]. A higher transfusion threshold (> 9 g/dL) was not associated with reduced risks of SAE or mortality [23, 24]. According to the current guidelines for adults with septic shock, the control of some early systemic insults are recommended as an initial target: mean blood pressure target of 65 mmHg over higher MAP targets [15] or the initiation of insulin therapy at a glucose level of ≥ 180 mg/dL (10 mmol/L) [25]. Because reported data on neurological status, particularly with respect to SAE, are scarce, the association of early systemic insults with outcomes in patients with SAE requires further investigations.

In the present study, we aimed to investigate the association of early systemic insults with outcome of critically ill patients with severe SAE. Specifically, we assessed early systemic insults within 48 h of ICU admission and the association of their correction on day 3 with 28-day mortality and awakening.

## Material and methods

### Patients

This study used the data from the French prospective multicenter OUTCOMEREA database (*n* = 12 ICUs) on patients enrolled between 1997 and 2020. OUTCOMEREA has already been extensively described elsewhere [26]. We included patients admitted to the ICU for at least 3 days with (a) severe SAE defined by a GCS ≤ 13 without sedation within the 24 first hours of ICU admission and (b) severe sepsis (defined by a systemic inflammatory response syndrome associated with an infectious episode and organ failure) or septic shock (defined as a sepsis associated at a low blood pressure persisting despite an adequate fluid resuscitation) using the prospectively collected data from OUTCOMEREA database [27]. For ease of analysis over the years, the SEPSIS 2.0 criteria have been used instead of the SEPSIS 3 criteria. For sedated patients, the last known GCS before sedation was used. We excluded patients admitted to the ICU with primary brain injury (including primary stroke), patients with an ICU length of stay less than three days (for analyses issues), patients with missing data on early systemic insults and outcomes (when measurement was not available, for analyses issues), patients with a limitation of life support from a collective decision in the first 48 h after ICU admission (to avoid biases on the studied outcomes) and patients without invasive ventilation in the first 48 h after ICU admission (to select the more relevant patients, e.g., difficulties to control systemic insults such as capnia if spontaneous ventilation). We assessed organ failure using the Sequential Organ Failure Assessment (SOFA) score [28] and the severity of the patients on ICU admission using the Simplified Acute Physiology Score 2 (SAPS II) [29].

### Systemic causes of secondary brain injury

For each patient, we analyzed the occurrence of the following early systemic insults during the first 48 h of ICU admission: hypernatremia (> 145 mmol/l), hyponatremia (< 135 mmol/l), hyperglycemia (> 11 mmol/l), hypoglycemia (< 3 mmol/l), hypotension (diastolic blood pressure < 50 mmHg), hyperthermia (T°C ≥ 38.3 °C), hypothermia (T°C < 36 °C), hyperoxia (PaO_2_ > 200 mmHg), hypoxemia (PaO_2_ < 60 mmHg), hypercapnia (PaCO_2_ > 45 mmHg), hypocapnia (PaCO_2_ < 35 mmHg) and anemia (hematocrit (Ht) < 21%). These data were collected at least once a day; if several measurements were performed on the same day, the worst value was used.

### Outcomes

We looked separately at the association of each early systemic insult within the first 48 h of ICU admission with outcomes, defined as mortality and awakening at day 28. We defined awakening as a GCS > 13 on two consecutive days within 28 days of ICU admission. If the patient was discharged before day 28, we used the value of the GCS value on the day of discharge. If the patient died within 28 days after ICU admission, we considered them as not awake at day 28. We also examined the association of the persistent systemic insults at day 3 with day-28 mortality and awakening. The GCS was collected at least once a day; if several measurements were performed on the same day, the worst value was used.

### Statistical analysis

Data are presented as numbers and percentages or medians and interquartile ranges. To identify the variables associated with better survival, we analyzed the presence of early systemic insults within the first 48 h of ICU admission and the effect of their correction on day 3 on outcomes at day 28. The primary endpoint was day-28 mortality. The secondary endpoint was awakening within 28 days following ICU admission. We performed a separate adjusted analysis on the association of each early systemic insult with day-28 mortality using a Cox model with adjusted Hazard ratio (aHR) and 95% confidence interval (95% CI) and with day-28 awakening using a multivariate analysis with a logistic regression model with adjusted odds ratio (aOR) and 95% confidence interval, ensuring that the influence of individual systemic insults could be studied separately. The aHRs and aORs were calculated after adjustment for the type of admission (medical versus other), the existence of hepatic comorbidities and the non-neurologic and non-hemodynamic components of the SOFA score to account for systemic organ dysfunction while avoiding redundancy and confounding. The hemodynamic component was excluded because hypotension was analyzed independently as a systemic insult, and the neurologic component was excluded as all patients had neurological dysfunction by design, defined by altered GCS. Statistical analyses used SAS 9.4. A p < 0.05 was considered statistically significant.

## Results

### Patients

**Fig. 1:**
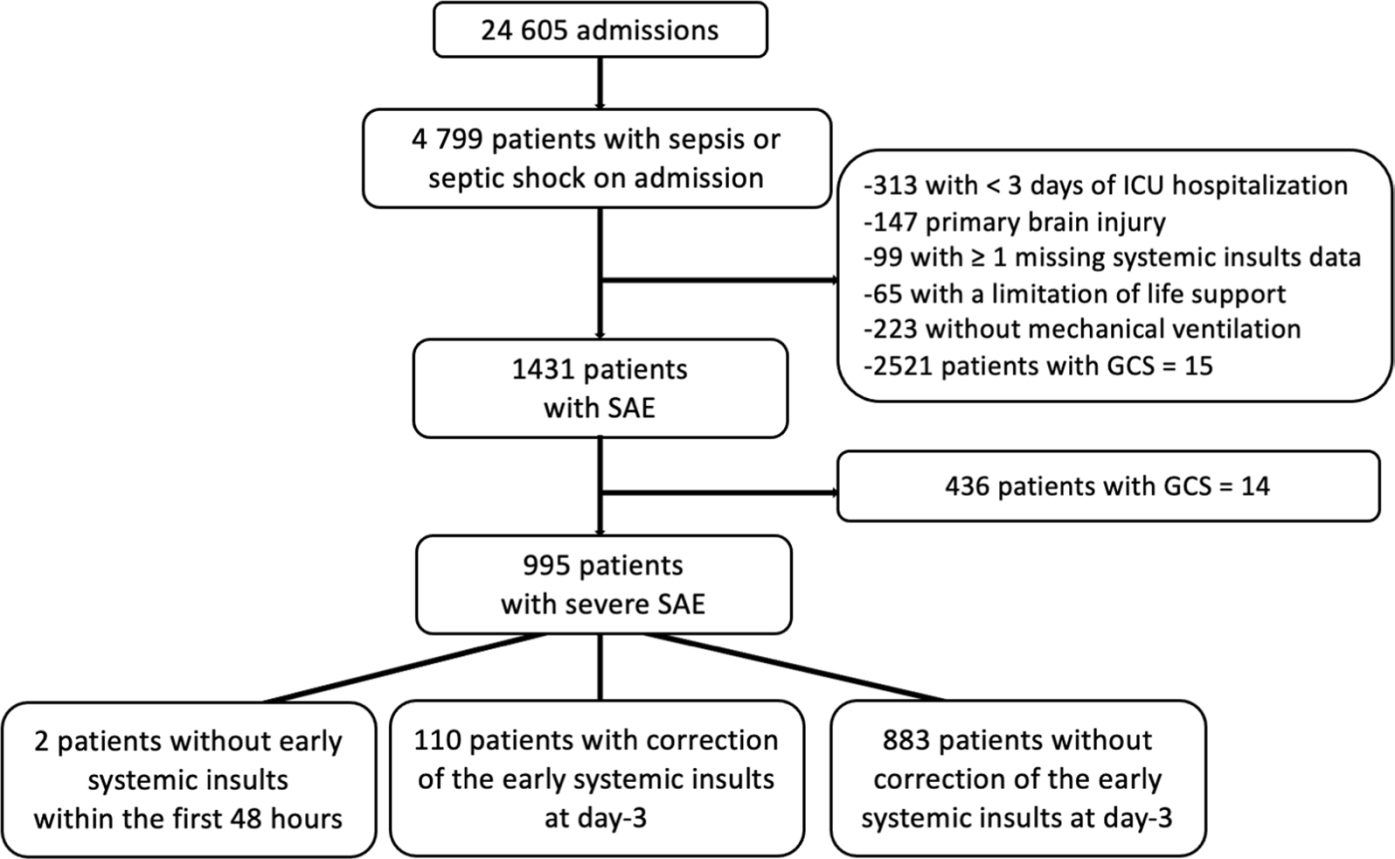
Flowchart. *GCS* Glasgow Coma Scale; *ICU* intensive care unit, *SAE* sepsis-associated encephalopathy

Among the 24,605 admissions during the study period, we identified 4799 patients with sepsis or septic shock at admission. We excluded 147 patients with primary brain injury, 313 patients who had a length of stay less than three days, 223 patients who did not receive invasive ventilation, 65 patients with life support limitations, 99 patients with missing data and 2521 patients with a GCS = 15. Among the remaining 1431 patients with SAE (GCS ≤ 14), we excluded 436 patients with a GCS score of 14 and finally included 995 (21%) patients with severe SAE (GCS ≤ 13) (Fig. 1). Baseline patient characteristics and their early systemic insults within the first 48 h are presented in Table 1. Patients were mainly males admitted for a medical condition, of whom 649 (65%) had a GCS ≤ 8. The most common comorbidities were cardiac and respiratory diseases, which were observed in 20% (*n* = 194) and 15% (*n* = 150) of cases, respectively, and 119 patients (20%) were immunocompromised. Sources and microorganisms from SAE episodes are shown in Supplemental Tables S1 and S2.

**Table 1:** Baseline characteristics and their association with day-28 mortality

Variable	Total	Survivors	Non-survivors	*p* value*
**At ICU admission**	*n* = 995	*n* = 599	*n* = 396	
Age, median [IQR]	68 [57.4–77.1]	66.7 [55.4–75.9]	69.8 [60.7–78.7]	< 0.001
Male sex, *n* (%)	636 (63.9)	372 (62.1)	264 (66.7)	0.226
Comorbidities, *n* (%)				
Chronic liver disease, *n* (%)	95 (9.5)	41 (6.8)	54 (13.6)	< 0.001
Chronic heart disease, *n* (%)	194 (19.5)	101 (16.9)	93 (23.5)	0.001
Chronic respiratory disease, *n* (%)	150 (15.1)	90 (15)	60 (15.2)	0.914
Chronic kidney disease, *n* (%)	79 (7.9)	42 (7)	37 (9.3)	0.131
Immunosuppression, *n* (%)	199 (20)	111 (18.5)	88 (22.2)	0.229
Alcoholism, *n* (%)	218 (21.9)	127 (21.2)	91 (23)	0.602
Smoking, *n* (%)	271 (27.2)	165 (27.5)	106 (26.8)	0.920
SAPS II, median [IQR]	67 [55–79]	63 [52–75]	73 [61–85]	< 0.001
SOFA without neurological, median [IQR]	9 [7–11]	10 [8–12]	13 [9–15]	< 0.001
Glasgow score, n (%)				
3–8	649 (65.2)	372 (62.1)	277 (69.9)	0.009
9–12	267 (26.8)	170 (28.4)	97 (24.5)	0.142
13	79 (7.9)	57 (9.5)	22 (5.6)	0.008
Medical admission, *n* (%)	698 (70.2)	401 (66.9)	297 (75)	0.007
Septic shock, *n* (%)	963 (96.8)	577 (96.3)	386 (97.5)	0.472
**Early systemic insults within the first 48 h**				
Glycemia				
Hypoglycemia (< 3 mmol/l), *n* (%)	63 (6.5)	23 (4)	40 (10.3)	< 0.001
Hyperglycemia (> 11 mmol/l), *n* (%)	333 (34.4)	199 (34.3)	134 (34.5)	0.969
Blood pressure				
Hypotension (DBP < 50 mmHg), *n* (%)	803 (80.7)	471 (78.6)	332 (83.8)	0.027
Temperature				
Hypothermia (T°C < 36 °C), *n* (%)	361 (36.3)	199 (33.2)	162 (40.9)	0.009
Hyperthermia (T°C ≥ 38.3 °C), *n* (%)	512 (51.5)	314 (52.4)	198 (50)	0.457
Anemia				
Hematocrit < 21%, *n* (%)	175 (17.7)	86 (14.4)	89 (22.6)	0.001
Natremia				
Hyponatremia (< 135 mmol/l), *n* (%)	350 (35.2)	209 (34.9)	141 (35.6)	0.808
Hypernatremia (> 145 mmol/l), *n* (%)	164 (16.5)	95 (15.9)	69 (17.4)	0.633
Oxygenation				
Hypoxemia (PaO_2_ < 60 mmHg), *n* (%)	126 (12.7)	67 (11.2)	59 (14.9)	0.070
Hyperoxia (PaO_2_ > 200 mmHg), *n* (%)	201 (20.2)	121 (20.2)	80 (20.2)	0.655
Capnia				
Hypocapnia (PaCO_2_ < 35 mmHg), *n* (%)	497 (50)	309 (51.7)	188 (47.5)	0.060
Hypercapnia (PaCO_2_ > 45 mmHg), *n* (%)	325 (32.7)	197 (32.9)	128 (32.3)	0.764

*We showed here the p-values from unadjusted Cox models

*IQR* interquartile, *SAPS II* Simplified Acute Physiology Score 2, *SOFA* Sepsis Related Organ Failure Assessment, *DBP* diastolic blood pressure

### ICU admission characteristics, early systemic insults, and outcomes

Univariate analyses investigating the association of baseline characteristics and early systemic insults with mortality and awakening are presented in Table 1 and Table 2, respectively. Within the first 48 h of ICU admission, all but two of the enrolled patients presented at least one early systemic insult. The most frequent early systemic insults were hypotension (n = 803, 81%), hyperthermia (*n* = 512, 52%) and hypocapnia (n = 497, 50%). In contrast, hypoglycemia (*n* = 63, 7%) and hypoxemia (*n* = 126, 13%) were less frequent. Hypoglycemia, hypotension, hypothermia, anemia within the first 48 h were associated with higher mortality on day 28. Hypoglycemia and anemia within the first 48 h were associated with less awakening on day 28.

**Table 2:** Baseline characteristics and their association with awakening on day 28

Variable	No awakening	Awakening	p value*
**At ICU admission**	*n* = 584	*n* = 411	
Age, median [IQR]	68.9 [58.8–78]	66.6 [55.8–76.2]	0.038
Male sex, *n* (%)	382 (65.4)	254 (61.8)	0.243
Comorbidities, *n* (%)			
Chronic liver disease, *n* (%)	64 (11)	31 (7.5)	0.073
Chronic heart disease, *n* (%)	128 (21.9)	66 (16.1)	0.022
Chronic respiratory disease, *n* (%)	93 (15.9)	57 (13.9)	0.373
Chronic kidney disease, *n* (%)	56 (9.6)	23 (5.6)	0.023
Immunosuppression, *n* (%)	134 (22.9)	65 (15.8)	0.006
Alcoholism, *n* (%)	146 (25)	125 (30.4)	0.059
Smoking, *n* (%)	5 (0.9)	9 (2.2)	0.090
SAPS II, median [IQR]	72 [59–84]	61 [51–73]	< 0.001
SOFA, median [IQR]	12 [9–14]	10 [8–12]	< 0.001
Glasgow score, *n* (%)			0.020
3–8	401 (68.7)	248 (60.3)	
9–12	144 (24.7)	123 (29.9)	
13	39 (6.7)	40 (9.7)	
Medical admission, *n* (%)	433 (74.1)	265 (64.5)	0.001
Septic shock, *n* (%)	570 (97.6)	393 (95.6)	0.086
**Early systemic insults within the first 48 h**			
Glycemia			
Hypoglycemia (< 3 mmol/l), n (%)	50 (8.8)	13 (3.3)	0.001
Hyperglycemia (> 11 mmol/l), n (%)	200 (35.1)	133 (33.3)	0.572
Blood pressure			
Hypotension (DBP < 50 mmHg), n (%)	479 (82)	324 (78.8)	0.210
Temperature			
Hypothermia (T°C < 36 °C), n (%)	222 (38)	139 (33.8)	0.176
Hyperthermia (T°C ≥ 38.3 °C), n (%)	303 (51.9)	209 (50.9)	0.748
Anemia			
Hematocrit < 21%, n (%)	121 (20.8)	54 (13.2)	0.002
Natremia			
Hyponatremia (< 135 mmol/l), n (%)	203 (34.8)	147 (35.8)	0.758
Hypernatremia (> 145 mmol/l), n (%)	103 (17.7)	61 (14.8)	0.238
Oxygenation			
Hypoxemia (PaO_2_ < 60 mmHg), n (%)	76 (13)	50 (12.2)	0.694
Hyperoxia (PaO_2_ > 200 mmHg), n (%)	113 (19.3)	88 (21.4)	0.425
Capnia			
Hypocapnia (PaCO_2_ < 35 mmHg), n (%)	182 (31.2)	133 (32.4)	0.689
Hypercapnia (PaCO_2_ > 45 mmHg), n (%)	284 (48.7)	213 (51.8)	0.932

*p-values from univariate logistic regression models

*IQR* interquartile, *SAPS II* Simplified Acute Physiology Score 2, *SOFA* Sepsis Related Organ Failure Assessment, *DBP* diastolic blood pressure

### Correction of systemic insults and outcomes

**Fig. 2:**
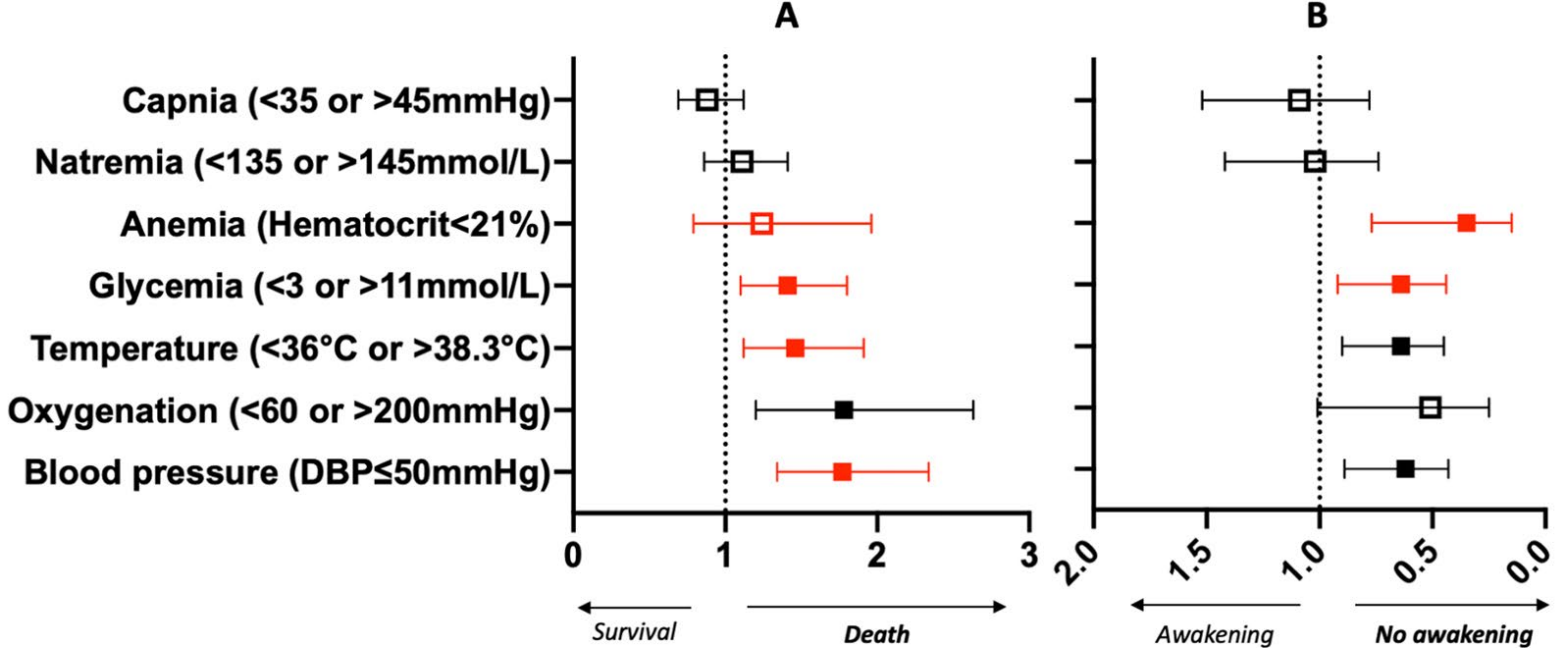
Forest plot of the multivariate analyses of the correction of the early systemic insults at day 3 on mortality and awakening on day 28. **A** Multivariate analyses of the correction of early systemic insults at day 3 on mortality on day 28. The aHRs are calculated after adjustment to the non-neurologic SOFA score (and exclusion of hemodynamic score for blood pressure) at ICU admission, the type of admission (medical versus other) and the existence of hepatic comorbidities. **B** Multivariate analyses of the correction of early systemic insults at day 3 on awakening on day 28. The aORs are calculated after adjustment to the non-neurologic SOFA score (and exclusion of hemodynamic score for blood pressure) at ICU admission, the type of admission (medical versus other) and the existence of hepatic comorbidities. Each line represents a separate adjusted analysis on the association of each early systemic insult with Day-28 mortality using a Cox model with adjusted hazard ratio (aHR) and 95% confidence interval [95% CI] and with Day-28 awakening using a multivariate analysis with a logistic regression model with adjusted Odds ratio (aOR) and 95% confidence interval [95% CI]. Red-colored square corresponds to a statistically significant early systemic insult associated to mortality and no awakening, respectively. A full square corresponds to a statistically significant persistent systemic insult associated to mortality and no awakening, respectively

Among the 995 patients with severe SAE, 883 (89%) exhibited at least one systemic insult that persisted through day 3. Persistent abnormal blood pressure (aHR = 0.77, 95% CI 1.34–2.34), oxygenation (aHR = 1.78, 95% CI 1.20–2.63]), temperature (aHR = 1.46, 95% CI 1.12–1.91) and glycemia (aHR = 1.41, 95% CI 1.10–1.80) at day 3 were independently associated with higher mortality (Fig. 2A).

Persistent abnormal blood pressure was independently associated with less day-28 awakening (aOR = 0.62, 95%CI 0.43–0.89) while a corrected blood pressure at day 3 was associated with higher day-28 awakening (aOR = 1.56, 95% CI 1.08–2.25). Persistent anemia (aOR = 0.35, 95% CI 0.15–0.77), abnormal temperature (aOR = 0.64, 95% CI 0.45–0.9) and glycemia (aOR = 0.64, 95% CI 0.44–0.92) at day 3 were independently associated with less awakening on day 28 (Fig. 2B). In contrast, we observed no such associations for natremia and PaCO_2_ levels.

Supplemental Table S3 shows adjusted analyses describing the association between the correction of each systemic insult at day 3 and day-28 mortality and awakening, respectively.

## Discussion

Our multicenter study provides prospective multicenter data regarding the incidence and potential adverse effects of early systemic insults in critically ill patients with severe SAE. Our findings indicate that 89% of patients with severe SAE exhibited at least one early systemic insult within the initial 48 h of admission to the ICU that persisted through day 3. We chose to analyze systemic insults on the third day (Day 3) because systemic insults on Day 1 primarily reflect the initial severity of the disease, while those on Day 3 represent the effects of therapeutic interventions and ICU management. Data beyond Day 3 were excluded due to the smaller sample size available after this point due to early mortality and missing data. Among this population, persistence of systemic insults—specifically, oxygenation, blood pressure, temperature, and glycemia—were independently associated with increased day-28 mortality. This study represents the first multicenter exploration revealing the independent association between persistent systemic insults and higher mortality rates in this patient cohort of patients with severe SAE.

Additionally, our investigation identified that several persistent systemic insults at day 3 (namely blood pressure, temperature, and glycemia) were independently associated with a reduced probability of awakening on day 28. These systemic insults may be subject to correction through appropriate medical intervention and care.

For systemic insults where no significant association with mortality or awakening on day 28 was observed, potential sensitivity issues may arise due to the limited availability of one value per day and the presence of potential confounding variables. In the case of natremia and capnia, we investigated the impact of more extreme thresholds (130 < *N* < 150 mmol/L and 30 < *N* < 50 mmHg for normal serum sodium and PaCO_2_ levels, respectively), producing consistent results.

The impact of systemic insults on SAE patients may have different effects depending on patients’ characteristics, and illness severity. For instance, natremia and PaCO_2_ may serve as primary contributors to SAE rather than true manifestations of systemic insults in the more severe patients. A previous study demonstrated that hypercapnia (PaCO_2_ > 45 mmHg) and hypernatremia (> 145 mmol/L) were independent risk factors for SAE [1]. Previous studies conducted in critically ill patients found that both hyponatremia (< 135 mmol/L) and hypernatremia (> 145 mmol/L) were independently associated with mortality [30–32]. A recent secondary data analysis from three large cohorts showed an association between serum sodium and 28-day mortality in sepsis patients with a U-shaped risk, increasing significantly below 137 and over 140 mmol/L [33]. Hypocapnia is a powerful cerebral vasoconstrictor which may induce cerebral ischemia and worsen brain injuries [34, 35]. A recent study showed an increased mortality in patients with suspected severe traumatic brain injury when end-tidal CO_2_ was below 35 mmHg [36]. Another multicenter study conducted in patients with traumatic brain injury, stroke or cardiac arrest found that hypercapnic acidosis, defined by a PaCO_2_ > 45 mmHg and a pH < 7.35, was also independently associated with mortality [4]. Conversely, a recent multicenter trial conducted in patients with coma who were resuscitated after out-of-hospital cardiac arrest showed that mild hypercapnia (PaCO_2_ between 50 to 55 mmHg) did not lead to better neurologic outcomes at 6 months than targeted normocapnia [37].

Our study has several limitations. First, a causal link between persistent systemic insults and mortality or awakening cannot be demonstrated with an observational design. We cannot exclude a substantial risk that the results are more general markers of disease severity than specifically markers of brain injury. We acknowledge that potential for Type I errors due to multiple testing. However, applying statistical corrections was not feasible in this study due to the limited sample size and the relatively low number of events, which could result in an overly conservative analysis and the loss of important signals. Second, the definition of SAE using GCS maybe confounded by several factors, including ongoing sedation and difficulties in the assessment of the verbal component in ventilated patients. In the literature, SAE is most frequently defined as an acute encephalopathy occurring during sepsis or septic shock, and not attributable to any other cause than sepsis itself which remains a broad syndrome with severity ranging from mild delirium to deep coma, impacting patient prognosis accordingly [38]. However, diagnosing SAE can be challenging without systematically ruling out status epilepticus (Convulsive and Nonconvulsive Status Epilepticus (CSE and NCSE)). Electroencephalogram (EEG), while sensitive for encephalopathy, is not specific to SAE, as similar patterns occur in other encephalopathies. Although the estimated incidence of status epilepticus in this population is 10–15% [39] with NCSE being rare in septic patients under continuous EEG monitoring [40], the absence of systematic EEG in our cohort represents a limitation unlikely to significantly affect our findings. Third, limitations of our data resolution (one value per day) may preclude the capture of rapid fluctuations in partial pressure of carbon dioxide (PaCO_2_) that, while not documented in the database, could wield a potentially significant influence. We used binary and not continuous variables for the correction of systemic insults because we had no details about duration of systemic insults such as hypotension per day. Fourth, patients with GCS 15 were excluded because delirium diagnosis was not documented in the database for these patients. This approach also follows previously established definitions, particularly those used in a 2017 study by Sonneville et al. [1]. Subsequently, patients with GCS 14 were excluded to focus on the most severe cases of encephalopathy and to avoid selection bias pointed out in a previous commentary [14]. Therefore, the prevalence of SAE in our cohort was lower than that observed in previous studies [1, 2, 14, 41, 42]. Fifth, due to the numerous potential confounders emerging after 3 days, we chose to focus our study within the first 3 days of ICU admission. We could have missed systemic insults developing later. Lastly, abnormal SSBIs manifesting after day 3 were not considered in our analysis. Consequently, the potential recurrence of systemic insults beyond this time frame could have exerted a substantial impact on the study outcomes.

## Conclusion

In severe SAE patients, early systemic insults were frequent within 48 h of ICU admission and the absence of their correction at day 3 was associated with increased mortality and decreased chances of awakening on day 28. Further interventional studies testing the impact of early correction of systemic insults in patients with SAE are needed to confirm these findings.

## Supplementary Information

Supplementary Material 1

## Data Availability

All data generated or analyzed during this study are included in this study or its supplementary material files. Further inquiries can be directed to the corresponding author.
