# Supplementary material for: Early systemic insults following severe sepsis-associated encephalopathy of critically ill patients: association with mortality and awakening—an analysis of the OUTCOMEREA database
Source: J Intensive Care. 2025 Jan 14;13:5. doi: 10.1186/s40560-024-00773-9 (PMC11730477; doi:10.1186/s40560-024-00773-9)
Supplement: Supplementary file 1 — Supplementary Material 1 [file 40560_2024_773_MOESM1_ESM.docx]

**Supplemental Material**

Article :

**Early Systemic Insults following Severe Sepsis-associated Encephalopathy of Critically Ill Patients: Association with Mortality and Awakening - an Analysis of the OUTCOMEREA Database.**

Table of contents:

-Supplemental Table S1: Sources of infection

-Supplemental Table S2: Microbiological documentation

-Supplemental Table S3: Multivariate analyzes of the control of early systemic insults at day 3 on mortality and awakening on day 28

**Supplemental Table S1 : Sources of infection**

| **Source of infection** | **n (%)** |
| --- | --- |
| Pneumonia | 477 (47.9) |
| Bacteremia | 236 (23.7) |
| Abdominal infection | 164 (16.5) |
| Urinary infection | 98 (9.8) |
| Surgical site infection | 66 (6.6) |
| Skin infection | 35 (3.5) |
| Catheter related infection | 18 (1.8) |
| Multiple sources | 235 (23.6) |
| Unknown | 72 (7.2) |

**Supplemental Table S2 : Microbiological documentation**

| **Microbiological documentation** | N=915 |
| --- | --- |
| Enterobacterales | 305 |
| *Pseudomonas aeruginosa* | 73 |
| Other non-fermentant | 5 |
| *Haemophilus* | 25 |
| *Staphylococci aureus* | 104 |
| Coagulase-negative Staphylococci | 35 |
| Enterococci | 67 |
| Streptococci | 119 |
| Pneumococci | 57 |
| Anaerobes | 30 |
| Other bacteria | 39 |
| *Candida* sp. | 59 |

**Supplemental Table S3: Multivariate analyzes of the control of early systemic insults at day 3 on mortality and awakening on day 28**

| **Variable** | **Outcomes** | | | | | |
| --- | --- | --- | --- | --- | --- | --- |
|  | **Day-28**  **Mortality** | | | **Day-28**  **Awakening** | | |
| **Early systemic insults at D3** | **aHR** | **[95% CI]** | **p** | **aOR** | **[95% CI]** | **p** |
| **Blood pressure (DBP>50mmHg)** |  |  | **<.001** |  |  | **<.001** |
| Normal within the first 48 hours | 1 |  |  | 1 |  |  |
| Controlled at day 3 | 0.76 | [0.55-1.04] |  | **1.56** | **[1.08-2.25]** |  |
| Not controlled at day 3 | **1.77** | **[1.34-2.34]** |  | **0.62** | **[0.43-0.89]** |  |
| **Oxygenation (60<N<200mmHg)** |  |  | **0.02** |  |  | **0.04** |
| Normal within the first 48 hours | 1 |  |  | 1 |  |  |
| Controlled at day 3 | 1.03 | [0.82-1.29] |  | 1.23 | [0.92-1.65] |  |
| Not controlled at day 3 | **1.78** | **[1.2-2.63]** |  | 0.51 | [0.25-1.01] |  |
| **Temperature (36°C<N<38.3°C)** |  |  | **0.007** |  |  | **0.01** |
| Normal within the first 48 hours | 1 |  |  | 1 |  |  |
| Controlled at day 3 | 1.11 | [0.84-1.47] |  | 0.95 | [0.68-1.33] |  |
| Not controlled at day 3 | **1.46** | **[1.12-1.91]** |  | **0.64** | **[0.45-0.9]** |  |
| **Glycemia (3<N<11mmol/L)** |  |  | **0.01** |  |  | **0.05** |
| Normal within the first 48 hours | 1 |  |  | 1 |  |  |
| Controlled at day 3 | 0.95 | [0.73-1.23] |  | 1.01 | [0.72-1.4] |  |
| Not controlled at day 3 | **1.41** | **[1.1-1.8]** |  | **0.64** | **[0.44-0.92]** |  |
| **Anemia (Hematocrit<21%)** |  |  | 0.16 |  |  | 0.18 |
| Not present within the first 48 hours | 1 |  |  | 1 |  |  |
| Controlled at day 3 | 1.27 | [0.97-1.66] |  | 0.76 | [0.51-1.14] |  |
| Present at day 3 | 1.24 | [0.79-1.96] |  | **0.35** | **[0.15-0.77]** |  |
| **Natremia (135<N<145mmol/L)** |  |  | 0.66 |  |  | 0.97 |
| Normal within the first 48 hours | 1 |  |  | 1 |  |  |
| Controlled at day 3 | 0.99 | [0.78-1.46] |  | 0.98 | [0.72-1.33] |  |
| Not controlled at day 3 | 1.11 | [0.86-1.41] |  | 1.02 | [0.74-1.42] |  |
| **Capnia (35<N<45mmHg)** |  |  | 0.36 |  |  | 0.55 |
| Normal within the first 48 hours | 1 |  |  | 1 |  |  |
| Controlled at day 3 | 0.82 | [0.63-1.08] |  | 1.22 | [0.85-1.77] |  |
| Not controlled at day 3 | 0.88 | [0.69-1.12] |  | 1.09 | [0.78-1.52] |  |

**Legend:**

Each cell represents a separate adjusted analysis on the association of each early systemic insults with Day-28 mortality using a Cox model with adjusted Hazard ratio (aHR) and 95% confidence interval [95% CI] and with Day-28 Awakening using a multivariate analysis with a logistic regression model with adjusted Odds ratio (aOR) and 95% confidence interval [95% CI]
The aHRs are calculated after adjustment to the non-neurologic SOFA score (and exclusion of hemodynamic score for blood pressure) at ICU admission, the type of admission (medical versus other) and the existence of hepatic comorbidities.

The aORs are calculated after adjustment to the non-neurologic SOFA score (and exclusion of hemodynamic score for blood pressure) at ICU admission, the type of admission (medical versus other) and the existence of hepatic comorbidities.

aHR: adjusted hazard ratio, aOR adjusted odds ratio, RBC: red blood cells.
